# Characterization and Determination of the Alkaloid Metabolites of *Evodiae fructus* in Rat Urine by Liquid Chromatography-Tandem Mass Spectrometry Detection

**DOI:** 10.3390/molecules16075822

**Published:** 2011-07-08

**Authors:** Rui Yan, Qier Mu, Yin Wang, Youping Liu, Xin Di

**Affiliations:** School of Pharmacy, Shenyang Pharmaceutical University, 103 Wenhua Road, Shenyang 110016, China

**Keywords:** metabolite, determination, liquid chromatography tandem mass spectrometry, *Evodiae fructus*, Zuojinwan preparation

## Abstract

The lack of authentic standards limits the quantitative analysis of herbal drugs in biological samples. This present work demonstrated a practicable assay of herbs and their metabolites independent of the availability of authentic standards. A liquid chromatography–electrospray ionization–mass spectrometry (LC–ESI–MS) method for the qualitative and quantitative determination of the metabolites after oral administration of *Evodiae fructus* and Zuojinwan preparation in rat urine has been developed. Urine samples extracted with a protein precipitation procedure were separated on a C_18_ column using a mixture of water (containing 0.1% formic acid) and acetonitrile (30:70, v/v) as mobile phase. The detection was performed by MS with electrospray ionization interface in positive selected reaction monitoring (SRM) mode. One urine sample after administration was selected as 'standard'. The method validation was carried out according to a conventional method that was calibrated by authentic standards. The fully validated method was applied to the pharmacokinetic study of the metabolites in rat urine. The results could provide evidence to explain the combination of *Coptidis rhizoma* and *Evodiae fructus* in terms of elimination.

## 1. Introduction

In Traditional Chinese Medicine (TCM), the combination of herbs often significantly alters their pharmacological properties. Studies on the interactions between herbs are useful for probing the mechanism of TCM [1]. Metabolite analysis is important for drug development. Due to the very low concentrations and complex matrix, metabolites are usually hard to separate from a biological system. As a result, previous studies on metabolites have always focused on qualitative analysis [2] and little information is available related to the quantities of metabolites. This paper presents an expedient method for the quantitative analysis of metabolites in biological samples. A urine sample obtained after administration acted as ‘standard’. With this method, we could assay the relative concentrations of metabolites in biological samples for comparative studies.

In TCM clinical practice, Zoujinwan preparation, which consists of *Evodiae fructus *- *Coptidis rhizoma* powder (1:6, g/g), has a long history of use to treat gastro-intestinal disorders [3]. Previous research on Zuojinwan preparation mainly focused on *Coptidis rhizoma *[4] while the function of *Evodiae fructus *has not been elucidated yet. *Evodiae fructus* is widely used in TCM due to its broad therapeutic effects, with evodiamine and its derivatives having been identified as the most important pharmacologically active constituents [5,6,7]. The disposition and metabolites of *Evodiae fructus* have been examined by several investigators using high performance liquid chromatography-tandem mass spectrometry [8,9,10]. As far as we know, no method for the simultaneous determination of metabolites in biological fluids after oral administration of *Evodiae fructus* has been published. Earlier publications have investigated the dissolution *in vitro*, pharmacokinetic and pharmacological properties of constituents in herbal combinations [1,11,12]. This paper will study the effects of combinations on the metabolism and excretion of metabolites.

## 2. Results and Discussion

### 2.1. Quality Analysis

**Figure 1:**
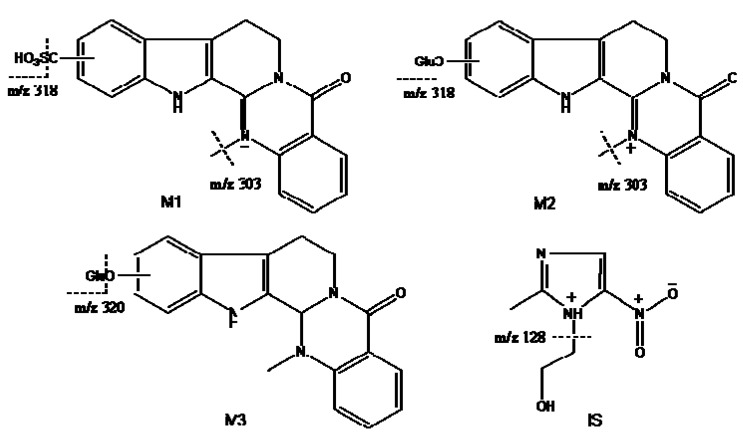
Chemical structure of metabolites and metronidazole (IS).

LC–MS^n^ was used for the qualitative analysis of the metabolites after administration of herb powder. The mobile phase was selected to optimize the separation and ionization efficiency. Three metabolites have not been reported previously and were found in both urine samples after administration of *Evodiae fructus* and Zuojinwan preparation. The molecular weights of the three metabolites were determined on the basis of their positive ion electrospray mass spectra, which showed precursor ions (Table 1). The possible structures of the metabolites were deduced by careful studies on their MS and MS^n^ spectra and by comparison with literature data [8,9,10,13]. The deduced chemical structures of three metabolites are shown in Figure 1.

**Table 1:** Precursor and product ions of metabolites in the rat urine in LC–MS^n^ experiments.

Metabolites	*m/z*	MS^2^	MS^3^	MS^4^	*t*_R_(min)	Formula	Identification
M1	398	318	303	275	17.44	C_19_H_16_N_3_O_5_S^+^	Hydroxydehydroevodiamine sulfate
M2	494	318	303	275	7.73	C_25_H_24_N_3_O_8_^+^	Dehydroevodiamine glucoronide
M3	496	320	187		19.69	C_25_H_25_N_3_O_8_	Hydroxyevodiamine glucoronide

### 2.2. Quantity Analysis

#### 2.2.1. Optimization of LC–MS/MS

**Figure 2:**
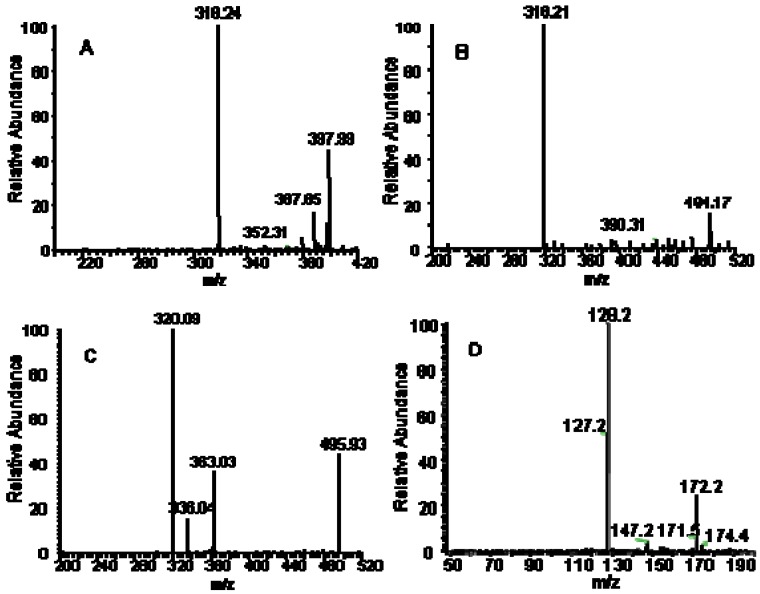
Product ion mass spectra of [M+H]^+^ ions of (A)M1 (B)M2 (C)M3 and (D)IS.

The typical MS^2^ full-scan ESI mass spectra of metabolites and IS is shown in Figure 2. The most abundant fragments in the product ion full-scan spectra of the substances were selected as the SRM transitions. To obtain maximum sensitivity of the SRM, some parameters such as spray voltage, capillary temperature, source CID, sheath gas (nitrogen) pressure, auxiliary gas (nitrogen) pressure, collision gas (argon) pressure, and collision energy were optimized. The other MS parameters were adopted from the recommended values for the instrument.

**Figure 3:**
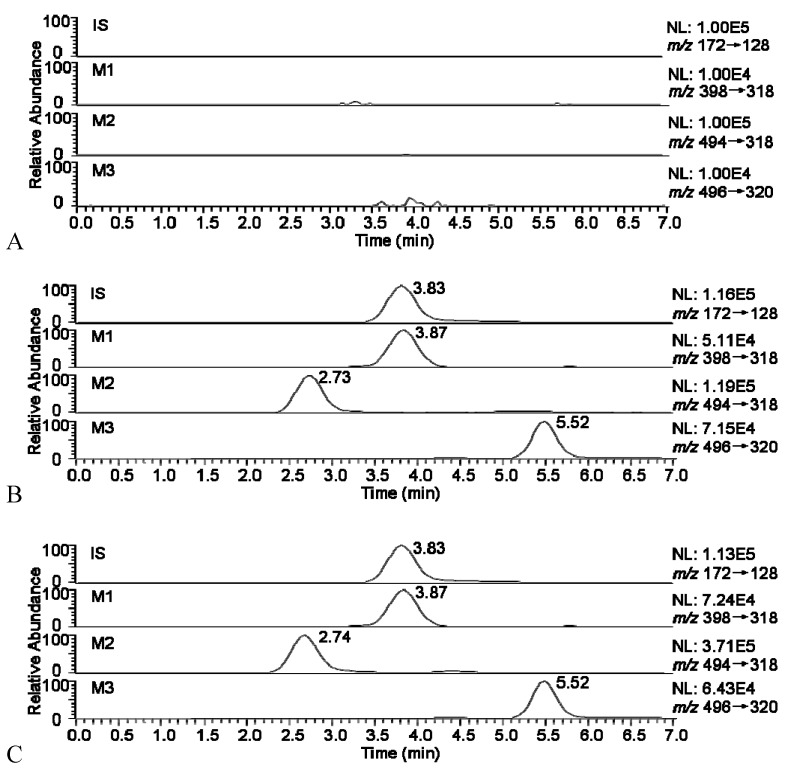
Representative SRM chromatograms for metabolites in a urine sample: (**A**) blank; (**B**) after administration of Zuojinwan preparation; (**C**) after administration of *Evodiae fructus*.

The selected mobile phase provided low background noise and suitable retention times. The typical chromatograms of blank urine and a urine sample 0~24 h after oral administration are presented in Figure 3. All samples were found to show no interference at the retention times of the analytes or the IS.

#### 2.2.2. Method validation

The sample preparation process may introduce errors in the determination which can be corrected with an internal standard method. As internal standard, metronidazole does not exist in the herb extract and functions through a different ion channel than the analytes. After separation by HPLC, the retention time of metronidazole fell in the middle of the analytes. In addition, its recovery rate was 81.5%, which was generally consistent with that of the analytes and thus ensured an ideal result.

All calibration curves showed excellent linearity over the range 0.025~1.000c in rat urine. Typical equations of the calibration curve using weighted (1/x^2^) least squares linear regression were as following: y = 0.00121+0.291x, *r*^2^ = 0.9871 (M1), y = −0.0209+3.524x, *r*^2^ = 0.9906 (M2), y = 0.0387 + 1.347x, *r*^2^ = 0.9933 (M3). The detection limits (LOD) of the three metabolites were all 0.005c. The precision and accuracy data corresponding to LLOQ are shown in Table 2.

**Table 2:** The precision and repeatability for the metabolites in rat urine (n = 6).

Analyte	Added	Found	RSD%		Relative error	Recovery %	
	(c)	(c)	Intra-day	Inter-day	%	mean	SD
M1	0.025	0.024	8.0	/	−4.7	/	/
	0.050	0.049	10.0	6.9	−1.4	81.7	7.3
	0.250	0.201	11.8	14.9	−19.5	84.1	7.7
	0.750	0.743	7.3	8.0	−0.9	89.5	11.6
M2	0.025	0.023	9.5	/	−6.6	/	
	0.050	0.038	14.9	12.6	−24.9	85.4	8.3
	0.250	0.214	11.8	14.8	−14.4	94.0	4.1
	0.750	0.733	9.3	13.4	−2.3	79.1	3.3
M3	0.025	0.026	7.9	/	5.9	/	
	0.050	0.045	8.3	16.8	−10.7	80.0	12.7
	0.250	0.229	14.4	9.6	−8.3	84.2	9.2
	0.750	0.692	9.5	15.2	−7.7	89.8	8.1

Table 2 contains the intra- and inter-day precision and accuracy data for the three metabolites. Most accuracy and precision values were within recommended limits. The relatively high RSD may be caused by excessive endogenous substances in biological samples, when the urine sample acted as standard. The average extraction recoveries determined for the three analytes were consistent, precise and repeatable. Data are shown in Table 2. The mean extraction recovery of the IS was 81.5 ± 6.0%.

Table 3 summarizes the stability data of the QC samples. The results showed that all the samples were stable during these tests and there were no stability-related problems were observed during the routine analysis of samples for the pharmacokinetic study.

The data displayed in Table 4 indicated that endogenous substances slightly affected the ionization of metabolites and IS under the present chromatographic and extraction conditions when the ESI interface was utilized. The low RSD value of absolute ME in six different sources of rat urine indicated that the relative ME for the analytes were minimal in this study.

**Table 3:** The stability for the constituents and metabolites in rat urine (n = 6).

Analyte	Concentration	Short-term			Three freeze-thaw			Post-preparative		
	(c)	Mean (c)	RSD%	RE%	Mean (c)	RSD%	RE%	Mean (c)	RSD%	RE%
M1	0.05	0.05	5.1	7.7	0.06	6.0	12.2	0.05	3.0	−4.8
	0.75	0.76	13.9	0.7	0.81	7.6	7.9	0.78	9.0	4.3
M2	0.05	0.05	4.1	−7.1	0.05	1.9	0.1	0.04	3.0	−10.7
	0.75	0.81	13.6	8.3	0.72	12.6	−3.7	0.73	13.5	−2.2
M3	0.05	0.05	11.0	0.2	0.05	9.5	3.5	0.05	4.9	−8.6
	0.75	0.73	11.7	−3.2	0.75	13.3	−0.4	0.73	12.7	−2.1

**Table 4:** The matrix effect for the metabolites and IS in rat urine (n = 6).

Analyte	Nominal concentration	Matrix effect %	RSD %
M1	0.25c	87.6	6.5
M2	0.25c	113.6	9.0
M3	0.25c	114.1	8.9
IS	200 ng/mL	109.4	10.2

### 2.3. Application to Pharmacokinetic Studies in Rats

The validated analytical method was applied to the assay of metabolites in rat urine after oral administration of *Evodiae fructus* and Zuojinwan preparation. The excretion amount for each analyte can be determined according to the following equation: the volume of the urine samples (mL) × determined concentration (c). The results are presented in Table 5.

**Table 5:** Contents of the metabolites in rat urine (n = 6).

Analyte	*Evodiae fructus* (c)				Zuojinwan preparation (c)			
	0~24 h		24~48 h		0~24 h		24~48 h	
	Mean	SD	Mean	SD	Mean	SD	Mean	SD
M1	10.03	0.75	0.54	0.12	2.09	0.61	0.35	0.01
M2	3.74	0.61	0.68	0.15	2.74	0.51	0.55	0.03
M3	5.27	0.11	1.27	0.27	5.45	0.11	1.96	0.23

More metabolites were eliminated within 0–24 h than within 24–48 h, which indicated a higher rate of elimination in 0–24 h than in 24–48 h; less *Evodiae fructus *metabolites were eliminated after combination with *Coptidis rhizoma* in Zuojinwan preparation than before, which suggested that *Coptidis rhizoma *might moderate the metabolism and elimination of the components in *Evodiae fructus*. As a result, *Coptidis rhizoma* plays the role of strengthening the effect of *Evodiae fructus* in Zuojinwan preparation [14]. One-way analysis of variance was applied to the comparison of the results before and after coupling: 0~24 h: F_0.05_(1,4) = 1.84 < 7.71, there is significant difference; 24~48 h: F_0.05_(1,4) = 0.05 < 7.71, there is significant difference. In conclusion, combination could significantly affect the elimination of the three metabolites within different periods.

## 3. Experimental

### 3.1. Chemicals and Reagents

Methanol, formic acid, acetonitrile were of chromatographic grade from the Yuwang Chemical Factory (Shandong, China). Deionized water for the preparation of samples and buffer solution was purified by use of an Alpha-Q water-purification system (Millipore, Bedford, MA, USA). All other reagents were of analytical grade. *Evodiae fructus *and *Coptidis rhizoma* were purchased from the Sifang Pharmacy (Shenyang, China). The contents of evodiamine and rutaecarpine were 0.62 and 0.94 mg/g *Evodiae fructus*, respectively, determined according to the Chinese Pharmacopeia.

### 3.2. Pharmacokinetic Study

Six male Sprague-Dawley rats (250 ± 20 g) were fasted for 12 h prior to experiment. The rats were split into two groups to complete the crossover design for pharmacokinetic experiment with a washout period of seven days. The powder of *Evodiae fructus* and Zuojinwan preparation was suspended in 0.1% carboxymethyl cellulose sodium (CMC-Na) aqueous solution and was administered to the rats (1.08 g *Coptidis rhizoma* and 0.18 g *Evodiae fructus* powder/kg body weight) by oral gavage. Urine samples were collected within 0~24 h and 24~48 h following administration, measured the volume of each sample accurately and stored at −20 °C for preservation.

### 3.3. Quality Analysis

#### 3.3.1. Apparatus and operating conditions

Qualitative analysis was operated on a Thermo-Electron LCQ linear ion-trap mass spectrometer (Thermo-Electron, San Jose, CA , USA) fitted with an electrospray ionization source over the mass range from *m/z* 50 to 2,000 in the positive ionization mode. The Xcalibur1.2 data analysis system was used. The spray voltage was set to 4.2 kV. The capillary voltage was fixed at 13 V. The heated capillary temperature was fixed at 200 °C. Nitrogen used as the sheath and the auxiliary gas was set to 70 and 20 arbitrary units, respectively. The isolation width for MS^n^ was 1.0 Da. The HPLC system consists of an Agilent 1100 series equipped with an Agilent 1100 series photodiode-array detector (PDA) and autosampler Data analysis (Agilent, Palo Alto, CA, USA). Chromatographic separation was carried out on a Diamonsil C_18_ (150 × 4.6 mm I.D., 5 µm, Dikma) with an EasyGuard C_18_ Security guard column (8 × 4.0 mm I.D., Dikma). The mobile phases consisted of 0.3% formic acid (A) and acetonitrile (B) using a gradient elution of 20% B at 0 min, 45% B at 25 min, 45% B at 40 min, at a flow rate of 0.5 mL/min. The column temperature was 30 °C, detection wavelength was at 245 nm and the injection volume was 20 μL.

#### 3.3.2. Sample preparation

One mL urine samples were filtered through 0.45 μm micro membrane (Truelab Co. Shanghai). The filtrate was passed through C_18_ solid-phase extraction cartridges (200 mg/3 mL) (Waters Co., Milford, MA, USA) that had been activated with MeOH (2 mL) and equilibrated with water (2 mL). The constituents were washed with water (1 mL) and eluted with MeOH (2 mL) from the cartridge, and then the eluate was evaporated under a stream of nitrogen at 45 °C to leave a residue that was dissolved in 200 μL of mobile for LC/MS^n^ analysis.

### 3.4. Quantitative Analysis

#### 3.4.1. Apparatus and operating conditions

The HPLC system consists of a LC-10ADvp Pump (Shimadzu, Kyoto, Japan) and a SIL-HTA Autosampler (Shimadzu, Kyoto, Japan). Chromatographic separation was carried out on a Diamonsil C_18_ (150 × 4.6 mm, 5 μm, Dikma) column with a EasyGuard C_18_ Security guard column (8 × 4.0 mm I.D., Dikma) kept at 20 °C. The mobile phase consists of water (containing 0.3% formic acid)/acetonitrile (30:70, v/v), at a flow rate of 0.45 mL/min. The injection volume was 10 μL.

Mass spectrometric detection was performed on a Thermo Finnigan TSQ Quantum triple quadrupole mass spectrometer (San Jose, CA , USA) equipped with an ESI source in the positive ionization mode. The MS operating conditions were optimized as follows: the spray voltage: 4,200 V; the heated capillary temperature: 320 °C; the sheath gas (nitrogen): 30 Arb; the auxiliary gas (nitrogen): 5 Arb; the collision gas (argon) pressure: 1.2 mTorr. Data acquisition was performed by Xcalibur 2.0 software. Peak integration and calibration were performed using LCquan software. Quantification was obtained by using SRM mode of the transitions at *m/z *398→318 for M1, at *m/z *494→318 for M2, at *m/z *496→320 for M3 and at *m/z *172→128 for metronidazole (IS) respectively, with a scan time of 0.3 s per transition.

#### 3.4.2. Standard solution and quality control samples preparation

No. 3 rat urine 0~24 h after oral administration of Zuojinwan preparation acted as standard stock urine with an apparent concentration of c for each analyte. Working solutions of the analytes were prepared by mixing 5, 10, 20, 50, 100, 150, 200 μL of the standard stock urine with 195, 190, 180, 150, 100, 0 μL blank rat urine, respectively to yield the following concentrations: 0.025, 0.05, 0.1, 0.25, 0.5, 0.75, 1.0c. Quality control (QC) samples at concentrations of 0.05, 0.25, 0.75c were prepared in the same manner. The working solution of internal standard (IS), at 200 ng/mL was prepared by diluting metronidazole stock solution (200 μg/mL) with methanol. All the solutions were stored at −20 °C.

#### 3.4.3. Sample preparation

To rat urine (200 μL) in a 1.0 mL Eppendorf tube, the internal standard solution (50 μL, 200 ng/mL) and acetonitrile (800 μL) were added. This mixture was vortex-mixed 2 min and centrifuged at 4,000 rpm for 5 min. The supernatant was separated out and blown to dryness with nitrogen at 40 °C. Then the residue was reconstituted in 100 μL mobile phase and mixed to make final testing samples. A 10 μL aliquot of the final testing samples was injected onto the LC−MS/MS system for analysis.

#### 3.4.4. Method validation

The method was validated according to the currently accepted USA Food and Drug Administration (FDA) bioanalytical method validation guidance [15]. The selectivity was investigated by preparing and analyzing six different batches of blank rat urine samples to ensure the absence of endogenous compounds with the same retention times as metabolites and internal standard.

The analytes’ calibration curve was generated by plotting the peak-area ratios of analytes to the IS (y) versus the concentrations of analytes (x), using weighed least squares linear regression (the weighing factor was 1/x^2^). The LLOQ for each analyte in urine, was defined as the lowest concentration at which both precision and accuracy were less than or equal to 20%.

Accuracy and precision were investigated by determining LLOQ and QC samples at three concentration levels of 0.025, 0.05, 0.25, 0.75c (six samples for each concentration level) on three different validation days. The concentration of each sample was calculated using a calibration curve constructed on the same testing day. Accuracy was described as relative error (RE) and precision was described as relative standard deviation (RSD). The criteria used to assess the suitability of precision and accuracy was as follows: the RSD should not exceed 15% and the accuracy should be within 15% of the actual value for QC samples.

To determine extraction recovery, extracted samples were prepared as the following procedure: QC samples at three concentration levels of 0.05, 0.25, 0.75c (three samples for each concentration level) were processed according to the “Sample preparation”; half of these processed QC samples were reconstituted in blank urine (200 μL), and then processed according to the “Sample preparation”. The extraction recoveries of the analytes were determined by comparing the mean peak areas of six re-extracted low (0.05c), medium (0.25c) and high (0.75c) samples to mean peak areas of six extracted samples at the same concentrations. Recovery of IS was also evaluated by comparing the mean peak areas of six extracted medium samples to mean peak areas of six reference solutions spiked in extracted plasma samples of the same concentration.

By exposing QC samples to different temperature conditions for different periods of time, the stability of analytes was investigated at two concentration levels of 0.05 and 0.75c (three samples for each concentration level). The stability studies included: (a) stability at room temperature for 4 h; (b) stability after three freeze–thaw cycles; (c) stability of the extracted samples at room temperature for 24 h.

The matrix effect (ME) was examined by comparing the peak areas of the metabolites between two different sets of samples. In set 1, QC at middle concentration was processed according to “Sample preparation”. These analyses were repeated six times. In set 2, six different batches of blank rat urine samples were processed according to the “Sample preparation”. The residue was reconstituted in stock standard urine (50 μL) and blank urine (150 μL). The mixture was processed according to “Sample preparation”. Ratio of the mean peak areas of set 2 to that of set 1 would indicate the possibility of ionization suppression or enhancement for analytes and IS. If the ratio is less than 85% or more than 115%, an exogenous matrix effect is implied. The assessment of the relative ME was made by a direct comparison of the analyte peak area values between different sources of urine. The ME of internal standard was assessed by comparing the peak area of its working solution added into the extract of precipitated blank urine with the peak area of the working solution.

## 4. Conclusions

Metabolism is extremely important in clinical studies. The ability of the assay to simultaneously quantitate metabolites can provide valuable information regarding the concentration versus time profiles to explain the formulation, the most appropriate dose and route of administration for TCMs. In order to satisfy the urgent demands for quantitating method of metabolites in the medicinal study, we established a simple, rapid and sensitive LC–MS/MS assay for simultaneous determination of the metabolites of *Evodiae fructus* and its preparation in rat urine.

The urine of rats after oral administration TCM was analyzed by LC-MS^n^. The structural information of three metabolites was deduced based on their MS^n^ spectra. A LC–MS/MS assay has been developed for the quantitative analysis of them in rat urine for the first time. The assay was applied to the pharmacokinetic studies of the metabolites in urine. The results can help to analyze the combination of *Coptidis rhizoma *and *Evodiae fructus* in Zuojinwan preparation.
